# Framing the Canadian Health Technology Assessment as an Organisational Process: A Bibliometric Analysis

**DOI:** 10.1002/hpm.70132

**Published:** 2026-09-05

**Authors:** Salvatore Tallarico, Simone Lazzini, Luisa Pellegrini, Harindra C. Wijeysundera

**Affiliations:** ^1^ Department of Economics and Management University of Pisa Pisa Province of Pisa Italy; ^2^ Institute of Health Policy, Management, and Evaluation University of Toronto Toronto Ontario Canada; ^3^ Department of Energy, Systems, Territory, and Construction Engineering University of Pisa Pisa Province of Pisa Italy

**Keywords:** bibliometric analysis, Canada, HTA

## Abstract

Health Technology Assessment (HTA) is an organizational process used to determine the value of health‐technologies. There is considerable variation in how HTA is developed globally, with Canada at the forefront of this field, having pioneered the use of HTA and implemented it extensively across healthcare organisations at all levels. This study is relevant for its unique focus on citation networks and author keywords regarding HTA in Canada, providing a holistic view by analysing HTA as an organizational process. By identifying fundamental process elements and differentiating research streams (RSs), it addresses a notable gap in the literature, offering fresh insights and informing policy recommendations to enhance HTA practices. While referring to the Canadian HTA, this study conducts a Systematic Literature Network Analysis to identify the RSs that will be analysed according to a process perspective. This analysis allows highlighting the process components—like stakeholders, phases, and methods—that have played a role in the success of Canada's HTA, as well as pinpointing the ongoing challenges that need to be tackled to create greater value. Analysing documents published in peer‐reviewed English‐language journals from the Scopus‐Elsevier database, we identified seven RSs, including macro‐HTA, meso‐HTA, micro‐HTA, ethical considerations, and patient involvement. For each stream, this study delineates the process elements, that is, the involved stakeholders, process phases, methodologies, and evaluation criteria. It is evident that while expert panels and specialist groups are crucial for technical HTA activities, patients and caregivers play a pivotal role in social and ethical evaluations. The study also highlights future challenges for HTA procedures: integrating qualitative and quantitative considerations, assessing the real impact of macro‐HTA outputs, and overcoming barriers to patient involvement. Theoretically, this paper clarifies the differences between streams in terms of HTA process elements, and practically, it assists practitioners in tailoring the HTA process to their specific needs. Policymaking recommendations include considerations regarding the number and intensity of stakeholder engagement.

## Highlights

- Different Canadian HTA research streams show different HTA process elements.
- Macro‐HTA process presents numerous and well‐structured phases.
- Qualitative HTA evaluations requires the patients, caregivers, and citizens involvement.
- Hybrid evaluation methodologies are the most implemented in Micro/Meso‐HTA.
- Equity‐based cost‐effectiveness (C/E) analysis should overcome the traditional C/E analysis.

## 1 Introduction

In recent decades, healthcare organisations have faced significant challenges in evaluating innovative health‐technologies. The rapid pace of technological advancements has created a pressing need for timely assessments that align with patient care needs and the evolving landscape of biomedical technologies. Health Technology Assessment (HTA) has emerged as a multidisciplinary approach, evaluating the economic, clinical, and social impacts of introducing new technologies into healthcare systems [1].

Among the most developed countries, Canada stands out as a pioneer in HTA [2]. Existing research has delved into various aspects of HTA within Canada, addressing specific topics such as orphan drug pricing [3], ethical considerations [4], patient involvement [5], and reimbursement/coverage procedures [6]. However, these studies often focus on isolated components of the HTA process rather than providing a comprehensive analysis of HTA as an integrated organizational process, encompassing inputs, outputs, performance, controls, resources, phases, activities, interdependencies, and management methods [7]. This reveals a significant gap in the literature: a holistic view of HTA processes from a systemic perspective remains underexplored. Instead, using a process‐based approach in this literature analysis allows us to highlight key elements of the HTA process that contributed to the success of the Canadian HTA. Since our analysis focuses on the Canadian healthcare system, which is renowned for its excellence and serves as a benchmark in HTA [8], the use of a process‐based approach helps identify all the process elements that have contributed to this high standard. Understanding these elements is the first step for those aiming to ensure that the outcomes of the HTA process effectively meet the needs of end‐users, primarily patients, thereby creating value for them.

As stated above, previous reviews have addressed specific aspects in isolation rather than providing a comprehensive analysis of HTA as an organizational process and this has prevented the identification of what is useful to create a successful HTA system. This paper aims to fill this gap by examining HTA as an organizational process within current research streams (RSs)—defined as a series of related papers deeply investigating a specific topic.

Our research objectives are to answer the following Research Questions:

- RQ1: What are the most investigated Canadian HTA research streams?
- RQ2: What are the main elements of the HTA process across different Canadian HTA research streams?
- RQ3: What are the HTA research trends and objectives for future study?

To address these questions, we employed a Systematic Literature Network Analysis (SLNA), following the methodology outlined in [9]. Systematic literature review methodologies, including SLNA, synthesise scientific evidence transparently and reproducibly to answer specific research questions. These approaches critically evaluate published evidence, reducing bias and enhancing the reliability of findings, thus supporting evidence‐based decision‐making in fields like public policy and health sciences. Compared to traditional systematic reviews, the adoption of Science and Literature Network Analysis (SLNA) offers a more structured and dynamic perspective, enabling deeper insights into research evolution, current trends, and future directions [9]. While traditional reviews provide static overviews, SLNA maps connections between authors and papers, helping to interpret large literature bodies and organise them into coherent research programs—such as HTA in Canada.

SLNA also leverages bibliometric network analysis to objectively detect emerging trends and track the evolution of research areas over time, overcoming the limitations of conventional reviews. This dynamic approach helps identify research gaps, improvement areas, and ongoing challenges.

To implement SLNA, we sourced documents from peer‐reviewed journals indexed in Scopus‐Elsevier. Using a list of keywords with Boolean operators, we queried Scopus and selected relevant documents after reviewing abstracts and full papers. Analysis was conducted with VOSViewer and Pajek to ensure reproducibility. SLNA allowed us to address the research gap by first performing Citation Network Analysis (CNA) to categorise current research on HTA topics. Next, Main Path Analysis explored foundational research traditions and interconnections between network clusters identified by VOSViewer. Finally, we examined papers through an organizational process‐analysis lens to identify components defining HTA RSs.

This study offers both theoretical and practical contributions to the HTA field. By mapping RSs through a process‐oriented lens, it deepens understanding of Canadian HTA and identifies key research gaps and challenges. The findings provide HTA practitioners with valuable insights into core process elements—stakeholder roles, methodologies, and evaluation criteria—to support more informed decision‐making. Policymakers can also use these insights to strengthen the rigour, transparency, and inclusiveness of HTA policies.

The paper is organised as follows: after presenting the theoretical background, the subsequent sections will detail the methodology, including the application of SLNA and Main Path Analysis, followed by the results, discussion and contributions.

## 2 Theoretical Background

HTA is a multidisciplinary process for evaluating the impact of health‐technologies. It supports policy decisions and optimises resource allocation within healthcare systems [10]. By integrating medical, economic, organizational, social, and ethical dimensions, HTA provides a comprehensive framework for evidence‐based decision‐making [11].

Its implementation depends on factors such as resource availability, legal and regulatory frameworks, stakeholder engagement, and international collaboration—crucial in resource‐limited settings [12].

A key challenge is standardizing HTA processes [13]. Proposed frameworks for guiding HTA implementation and supporting policy‐making often fail because they overlook HTA as an overall organizational process and focus on specific issues.

Furthermore, stakeholder participation—particularly from patients/citizens—remains underdeveloped. Enhancing participation can improve the legitimacy and relevance of HTA outcomes [14].

Bibliometric analyses have examined trends in HTA research, with a focus on methodologies, stakeholder roles, and emerging technologies such as AI for diagnostics and patient monitoring [15] Similarly, methods like multicriteria decision analysis (MCDA) have been explored for their potential in guiding decisions in areas such as big data and telemedicine [16]. However, HTA is still rarely studied as a holistic organizational process. Despite calls for greater coordination in HTA [17], a comprehensive analysis of HTA as a structured process remains lacking.

Such analysis offers a comprehensive framework through which the above challenges can be addressed.

### 2.1 The Canadian Context

HTA in Canada is a multifaceted process that reflects the decentralised nature of the country's healthcare system. At the national level, the Canadian Agency for Drugs and Technologies in Health (CADTH) focuses on nationally significant technologies such as drugs and medical devices [18]. CADTH collaborates with specially convened advisory committees to assess HTA submissions, evaluating them based on of national priority for clinical effectiveness and cost. CADTH's Common Drug Review (CDR) evaluates new drugs to provide recommendations for inclusion in public drug coverage plans, aiming to minimise assessments duplication and ensure consistency and quality in coverage decisions [6]. At the provincial level, Alberta and Ontario have established HTA units to address specific local needs with more targeted HTA capabilities [1]. These activities reflect the challenges/opportunities of Canada's decentralised healthcare system, where timely assessments and transparent decisions remain priorities to enhance access and effectiveness of healthcare technologies nationwide.

The above‐described context stems from decades of HTA implementation in Canada where HTA is an established process [8] across various healthcare fields and levels. Previous works in Canada have considered specific aspects: orphan drug prices [3], ethical considerations [2], HTA reports analyses [8], patient involvement [19], MCDA use [20], and reimbursement procedures [6]. While valuable, these studies have examined only certain elements of the HTA process, often comparing different HTA methodologies, evaluation criteria, and levels of stakeholder involvement. For instance, previous authors conducted a review of HTA tools [21], international survey of policy/clinical‐decision‐makers regarding criteria usage, and highlighted variables influencing stakeholder involvement. Other contributions have assessed the efficiency and practical feasibility of HTA processes or emphasised the mechanisms by which HTA agencies achieve their overall impact.

However, none of these contributions have explored HTA as an organizational process, identifying its main components and distinguishing them across various RSs. Therefore, we address this gap introducing new trends and objectives within HTA.

## 3 Methodology

### 3.1 Structured Literature Networks Analysis (SLNA)

Using Elsevier's Scopus database, we employed the SLNA methodology Colicchia and Strozzi proposed [9], including: (1) a systematic literature review (SLR) to identify relevant papers, (2) citation network analysis (CNA) to visualise and study the networks, and (3) main path analysis to select papers forming the backbone of research traditions. Additionally, we conducted a keyword analysis, incorporating articles CNA excluded.

The first author compiled a list of potential keywords for (1), and all co‐authors selected the final list, defining a strategy for identifying relevant articles. Then the first author conducted (2) and (3) using VOSViewer and Pajek and proposed a thematic classification of RSs, which was subsequently refined through joint discussion to develop a shared understanding of key contents.

Given our research focus on understanding HTA as an organizational process and identifying its components across various RSs, SLNA is a well‐suited methodology. SLNA is effective for examining research domain evolution and establishing connections among authors, which helps in identifying RSs.

To implement the methodology effectively, we developed: extensive database searches, advanced Boolean searches for precision, document scanning to identify key themes, detailed notetaking, and structuring the review around emerging themes. This structured approach helped us integrate key concepts into a coherent narrative.

#### 3.1.1 SLR Analysis

The SLR aims to:

- Locate the studies.
- Select and evaluate the studies.

These steps output is the list of all relevant papers to be included in the CNA.

##### 3.1.1.1 Locating Studies

We compiled a preliminary list of keywords: ‘Health technology assessment’, ‘HTA’, and ‘biomedical technology assessment’ were identified as central to the study topic. ‘Canada’ was chosen given its established processes in this field. The remaining keywords (‘techniques’, ‘methodologies’, ‘stakeholders’, ‘actors’, ‘phases’, and ‘evaluation criteria’) aimed to explore HTA as an organizational process.

These keywords were reviewed, refined, and approved by colleagues who are experts in HTA and/or organizational processes, ensuring the validity of the selection. Boolean operators (AND, OR) were used to combine keywords, optimising search results and avoiding overly broad or irrelevant findings.

##### 3.1.1.2 Papers Selection and Evaluation

To ensure the substantive and empirical relevance of the selected papers, we applied the following criteria:

- Substantive Relevance: Only documents published in peer‐reviewed journals were included to guarantee scholarly rigour and information reliability.
- Empirical Relevance: We selected documents that explicitly mentioned the selected keywords in the title/abstract/keyword list.
- Field Alignment: Contributions not aligned with the HTA research field were excluded to ensure relevance.
- Language: Only documents published in English were considered to maintain consistency and accessibility.

After initial screening based on these criteria, we reviewed abstracts and concentrated on the promising documents. This thorough process ensured that only the most relevant papers were included, supporting the robustness and validity of our research findings.

We chose 311 papers as nodes for the CNA.

PRISMA framework (Figure 1) details the stages of documents' identification, screening, eligibility, and inclusion. Ultimately, 50 are the articles processed by VOSviewer to create citation and keyword networks, analysed hereafter.

**FIGURE 1:**
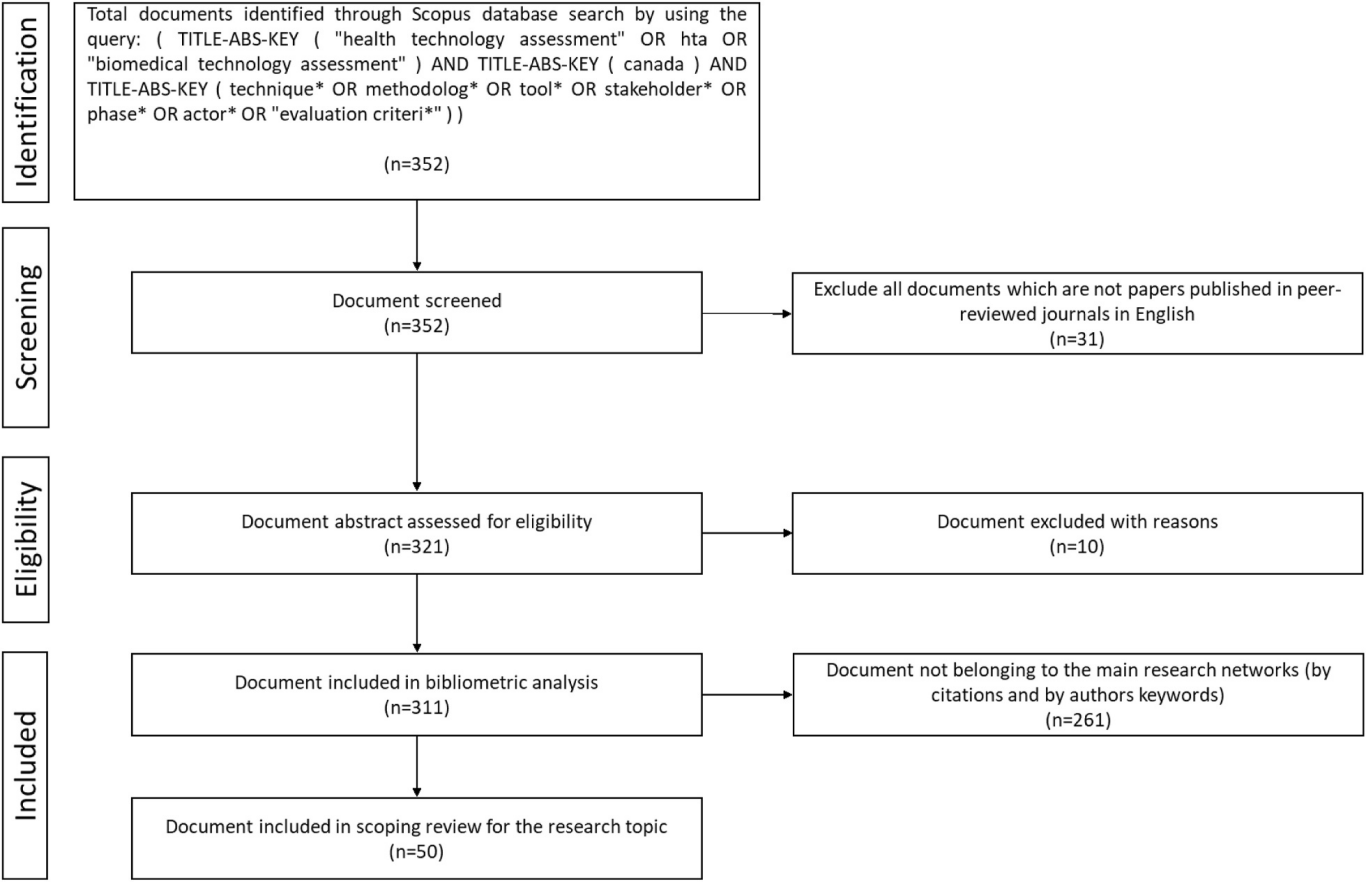
PRISMA framework.

#### 3.1.2 CNA Analysis

##### 3.1.2.1 Citation Networks

We first identified RSs that consist of related papers which explore specific topics, with citations reflecting their interconnectedness. We used Citation Network Analysis with VOSviewer to map these connections. This tool helps verify document links via citations and clusters articles into RSs, offering both static and dynamic views essential for a thorough literature understanding.

In a bibliometric network, nodes (documents) and edges (relationships) are visualised in a two‐dimensional space, where node distance indicates relatedness. VOSviewer uses distance‐based visualisation and colour‐codes clusters for easier RS identification. This method has been effectively applied in studies to map scholarly landscapes, ensuring a robust analysis aligned with our research objectives.

##### 3.1.2.2 Main Path

We performed main path analysis using Pajek, guided by VOSViewer results. Pajek helps extract and chronologically arrange the most cited articles, from oldest to newest. Documents are interconnected based on traversal frequency from the earliest to the most recent, creating a Main Path. This approach explores the evolution of theories or scholarly traditions relevant to a topic by tracing highly cited articles over time.

##### 3.1.2.3 Keywords Networks

To capture overlooked information, we conducted keyword network analysis using VOSviewer. Like citation analysis, this method involves nodes representing keywords and edges indicating co‐occurrences within papers. This analysis helps identify potentially significant but previously excluded information.

## 4 Results

### 4.1 Citation Networks

Figure 2 shows the network and its overlay visualisation by citations obtained importing in VOSViewer the 311 selected papers.

**FIGURE 2:**
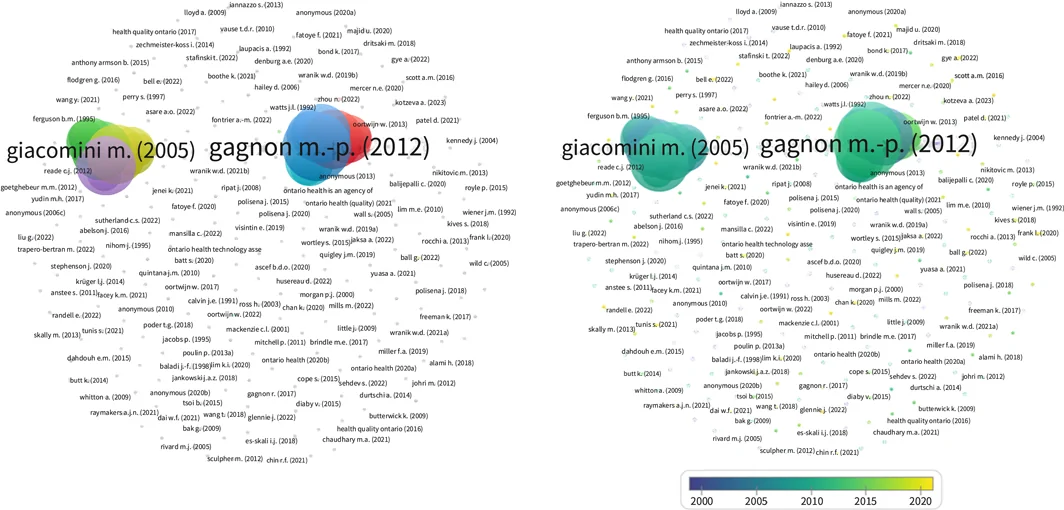
Citation network (left) and overlay citation network (right) visualisations.

Two main facts arise: (1) Most of the papers are not inter‐connected by citations, and there are two primary networks (a and b) composed of 8 articles each (Figure 3). These networks represent the nodes linked by citations, upon which potential RSs on HTA are built. Documents not interconnected by citations are excluded from the analysis because they appear as independent research, and hence do not belong to a RS. (2) All the most recent papers do not have bidirectional connections by citations, and the two primary networks are composed of contributions dating back ten or more years ago.

**FIGURE 3:**
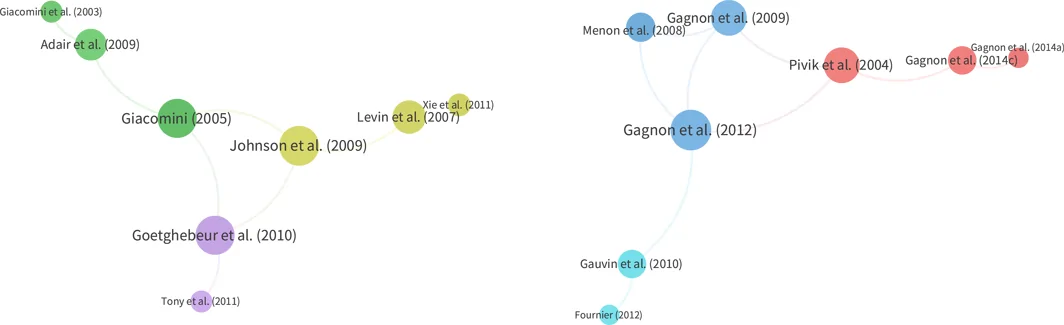
Primary networks (a on the left, b on the right).

Network (a) is composed of three clusters: (*a.1*) is focused on the ethical aspects, above all related to genetic issues, to be considered in the HTA process [6, 10, 22]; (*a.2*) regards the evidence‐based HTA [1, 23]; (*a.3*) is related to the use of the MCDA for supporting HTA [11, 20].

Network (b) is composed of three clusters: (*b.1*) and (*b.2*) talk about the patients' involvement, respectively, in the Hospital‐Based HTA [12, 24, 25] and in the early‐HTA [5, 19, 26]; (*b.3*) regards the importance of HTA outputs' dissemination [27, 28].

Table 1 briefly describes the two networks and the key papers that drive the topic for each cluster.

**TABLE 1:** Key paper driving the cluster topics within the networks.

Network	Cluster	Description	Key paper
a.	a.1	Network a reflects the need for a structured approach to decision‐making in HTA, underscoring the importance of building on methodologies to balance ethics and evidence. Ethical considerations (a.1) are crucial, particularly in sensitive fields like genetics, but must be integrated with robust evidence (a.2) to ensure scientifically sound outcomes. MCDA (a.3) offers a bridge to combine ethical values with empirical data in multi‐criteria evaluations.	[6] This article examines the ethical challenges health plans face when deciding whether to cover technologies with complex purposes, effects, or costs. It emphasises the integration of quantitative evidence and qualitative, ethical assessments. The study highlights key aspects of the decision‐making process, focusing on three stages: Reviewing past decisions and establishing ethical precedents, making decisions on new cases using ethical reasoning, and incorporating these decisions into an ongoing evaluation framework. These phases require specific ethical considerations, roles, and methods to guide policy application effectively.
	a.2		[23] The article describes the HTA approach, which takes a multidisciplinary perspective to assess safety, efficacy, economic and organizational impact, as well as social and ethical implications related to the adoption of technologies in healthcare systems. It focuses on the application of HTA in Canada, a pioneer in using this tool to support evidence‐based decision‐making. The article highlights federal and Ontario initiatives, with a focus on the collaboration between academic researchers and decision‐makers. It also addresses issues and challenges, encouraging broader discussion among HTA stakeholders to advance its progress.
	a.3		[11] This article discusses a study that tested a decision support framework (EVIDEM) for healthcare decision‐making, combining multicriteria decision analysis (MCDA) and health technology assessment (HTA). The study aimed to assess the framework's utility and reliability, using tramadol for chronic non‐cancer pain as a case study. The drug advisory committee evaluated the framework through workshop sessions, assigning weights, scores, and assessing qualitative impacts for various criteria. The results showed that the framework helped systematically consider a broad range of decision criteria and supported consistent decision‐making, though data limitations occasionally hindered its use. The approach demonstrated fair to good reliability and highlighted the need for further validation and testing to advance MCDA in healthcare decision‐making.
b.	b.1	Network b emphasises the growing importance of patient (early) engagement and communication. In hospital‐based HTA (b.1), involving patients helps align decisions with real‐world needs. Early‐HTA (b.2) extends this involvement to the initial stages of technology development, fostering a more patient‐centred approach. To ensure meaningful impact, HTA outputs must also be clearly communicated (b.3), promoting transparency and accessibility for all stakeholders	[24] This paper explores the need for greater patient involvement in HTA, particularly at the local level in Quebec, Canada. It focuses on a project where patients are engaged in an HTA evaluating alternatives to isolation and restraint for hospitalised adults. The paper outlines strategies for patient involvement, such as direct participation in the HTA process, consultations with patients and families, and involvement in disseminating results. It aims to assess the impact of these strategies on the relevance, acceptability, and sustainability of HTA recommendations, contributing to the broader knowledge of patient involvement in HTA.
	b.2		[5] This paper examines the growing interest in involving consumers in decisions related to HTA, which influences access to medical treatments. The study empirically assesses consumer interest in HTA involvement by identifying what health consumer organisations consider meaningful participation. It reviews current international practices and proposes a model for consumer involvement based on priorities and needs. The paper highlights that canadian health consumer groups, particularly those representing major disease conditions, expressed a strong desire to participate in HTA and provided feedback on potential mechanisms to facilitate their involvement.
	b.3		[28] The article highlights the importance of dissemination in the context of public involvement in health technology assessment (HTA). Effective dissemination is crucial for ensuring that the insights and feedback from the public are properly communicated to relevant stakeholders, including policymakers and healthcare professionals. The study suggests that HTA agencies must balance scientific and methodological standards with the need to convey public input in a way that influences decision‐making processes. By developing a conceptual tool to analyse public involvement, the article emphasises the role of dissemination in ensuring that diverse public perspectives are integrated into HTA processes, ultimately improving the transparency, inclusivity, and effectiveness of healthcare policies.

### 4.2 Main Path

Pajek was used for Main Path Analysis to determine if the primary network papers identified by VOSviewer represent the core of HTA traditions. This analysis clarifies the interconnections between clusters and identifies HTA RSs. Figure 4 displays the resulting paths:

**FIGURE 4:**
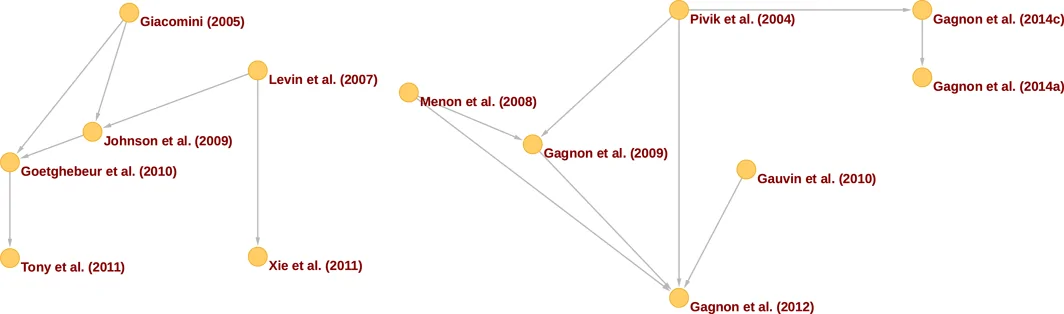
Main paths (ax on the left, bx on the right).

Path ax, comprising six articles, outlines the theory development leading to the MCDA use in HTA. MCDA integrates qualitative and quantitative criteria, including ethical aspects, to support evidence‐based decisions [1, 6, 20].

Path bx, comprising seven articles, explores the patient involvement development in HTA, especially in local HTA contexts. It traces the evolution from viewing patients as consumers to involving them as decision‐makers, highlighting increased public value with greater patient engagement [5, 12, 24].

Table 2 shows the main elements of the HTA processes within the RSs of paths.

**TABLE 2:** Primary networks clusters and main path analysis results.

Clusters		Research main paths		Elements of the HTA process				Literature gap(s)
Number	Focus	#	Description	Stakeholders	Phases	Methodologies	Evaluation criteria	
a.1	Ethics in HTA processes (about genetic issues) [6, 10, 22]	ax	Complex evaluations (not enough evidence to support decisional processes), that require to balance both qualitative and quantitative criteria. Need to introduce ethical criteria.	Hospitals, physicians, academic institutions, clinical epidemiologists, health economists, industry, and bioethicists	1. Taking stock of past decisions 2. Formulating precedents 3. Deciding new cases 4. Assimilating decisions	Network meta‐analysis, MCDA, EVIDEM framework, qualitative tools for ethical and health‐system‐related components	Overall clinical benefit, consistency with societal and ethical values, value for money, feasibility of adoption into the health system, purpose, effectiveness, additional effects, unit cost, demand, cost‐effectiveness	How to: — Incorporate in the HTA approach pragmatic post‐marketing studies, human factors, and safety analyses. — Establish formalised interaction with a broad spectrum of potential end‐users for each technology. — Join qualitative and quantitative considerations in the HTA procedures.
a.2	Evidence‐based HTA [1, 23]							
a.3	Use of MCDA in HTA [11, 20]							
b.1	Patients' involvement in hospital‐based HTA [12, 24, 25]	bx	Local and hospital‐based HTA. Patients' involvement in evaluation processes.	Patients, physicians, health and social services professionals, public health insurance agency, specialist group, association of health centres, administrators, clinical manager, HTA producers	1. Selection of assessment topics 2. Assessment 3. Reporting 4. Diffusion	Cost‐effectiveness analysis, cost‐utility analysis	Efficacy, safety, cost and cost‐effectiveness, ethical, social, organizational, and economic implications, lack of alternative, completeness of data on adverse event, cost, potential to extend life with quality, potential to detect a condition which, if treated early, averts future costs, potential clinical benefit over existing treatments, potential to improve quality of life, potential for additional application, potential to benefit to a number of people.	How to overcome the current barriers related to the patient's involvement.
b.2	Patients' involvement in early‐HTA [5, 19, 26]							
b.3	HTA outputs' dissemination [27, 28]							

The RS of path ax involves complex evaluations of health‐technologies [1, 6, 20, 23]. It requires balancing qualitative (e.g., consistency with societal values [1]) and quantitative criteria (e.g., unit cost [22]). Stakeholders include hospitals, physicians, academies, epidemiologists, economists, industry, and bioethicists [1]. Methodologies include frameworks like EVIDEM, qualitative tools, and MCDA [11, 20]. HTA process phases are: reviewing past decisions, formulating precedents, deciding new cases, and assimilating decisions for future assessments. Although the latest article in path ax dates to 2011, it underscores ongoing challenges, such as integrating post‐marketing studies and engaging a broad stakeholder's range [29]. Recent literature emphasises the need to combine qualitative and quantitative criteria in evaluations [6].

The RS of path bx focuses on patient involvement in HTA, particularly in Hospital‐Based HTA [5, 25, 28, 30]. Unlike path ax, patients are central here, alongside health professionals, public agencies, and specialist groups [29]. While no specific methodologies emerged, criteria like efficacy, effectiveness, and quality‐of‐life suggest that cost‐effectiveness (C/E) and cost‐utility (C/U) analyses are relevant. Indexes such as the Incremental Cost Effectiveness Ratio (ICER) and the quality‐adjusted life year (QALY) are patient‐centric. HTA process phases include topic selection, assessment, reporting, and dissemination. Although the path halted in 2014, patient involvement remains a key topic, with challenges such as time, resources, recruitment, and complexity continuing to hinder progress [26, 31].

### 4.3 Keywords Networks

CNA reveals that many nodes are not interconnected, potentially omitting significant information for our aim. Relying solely on CNA may overlook valuable insights in not included papers.

Therefore, we conducted a Keywords Network Analysis (KNA) using VOSviewer to visualise keywords‐based networks (Figure 5).

**FIGURE 5:**
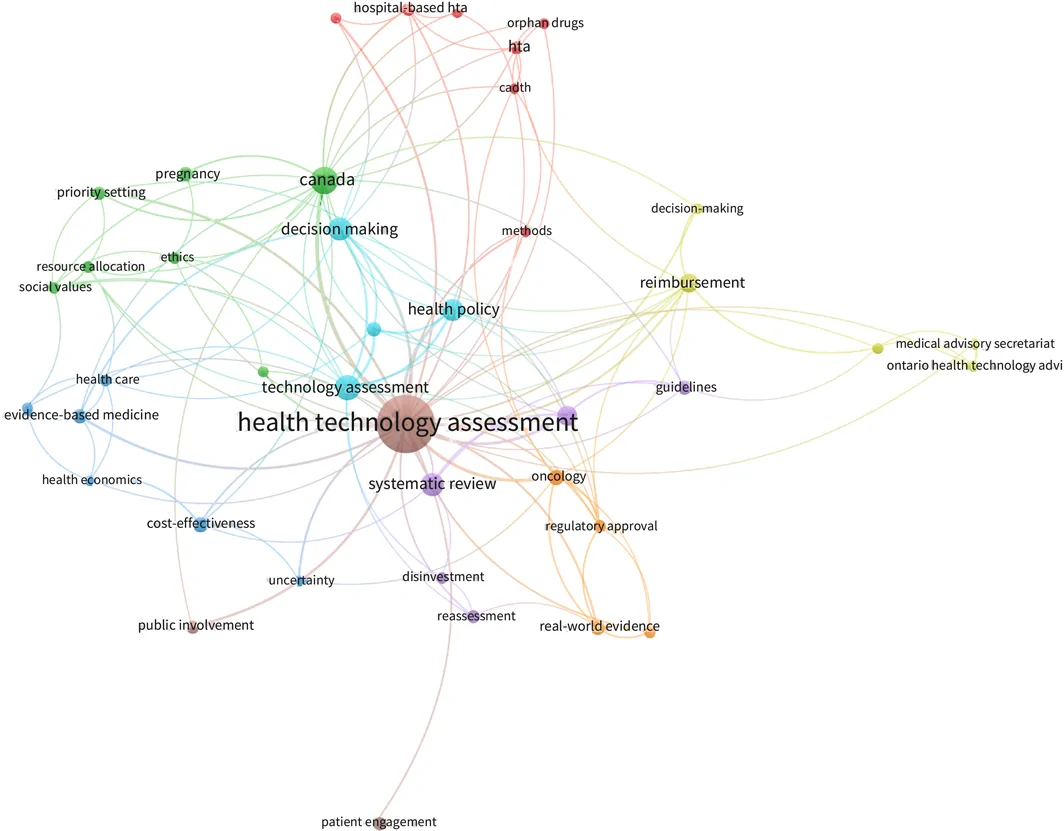
Keywords network visualisation.

The network comprises eight keyword clusters, detailed in the first two columns of Table 3. By integrating the outcomes from both CNA and KNA, we identified the RSs below described.

**TABLE 3:** Keywords network clusters analysis results.

Research stream			Elements of the HTA process				Literature gap(s)
Name	Keywords	Description	Stakeholders	Phases	Methodologies	Evaluation criteria	
Ethics in HTA processes	Ethics; health technology assessment [6, 18]	The HTA social value: How to consider ethics issues in evaluation processes	Patients, health system, clinicians, HTERP members, customers, experts, ethicists, HTA bodies, policymakers	1. Taking stock of past decisions and formulating precedents 2. Deciding new cases 3. Assimilating decisions	CADTH HTERP deliberative framework	Clinical benefit and harms (efficacy, safety, clinical management, non‐health benefits) Patient preferences (experiences with condition and technology) Economic impact (cost‐effectiveness, budget impact) Implementation (healthcare processes, work force) Legal impacts (legal or regulatory issues) Ethical issues (consistency with canadian values) Environmental impact (resource use, recyclability, waste disposal)	Defining ethical frameworks How to join qualitative and quantitative considerations in the HTA procedures
	Social value; health technology assessment [6, 30, 32]						
Economic analyses	Cost‐effectiveness; health economics [33, 34]	The use of cost‐effectiveness analysis as main methodology to conduct HTA evaluation (health economics reviews) under uncertainty	Specialist group, HTA agencies/bodies, HTA methodologists, Healthcare managers, health programme directors	1. Evaluation of the impact in terms of expected life years 2. Evaluation of the impact in terms of healthcare costs	C/E and C/U. Outcomes: QALY; ICER; LYG; years survived; relative effectiveness analysis	Costs, utility, effectiveness. To assess the technology value: Direct effects (benefits or harms) to patients; effects on clinical outcomes; management effects; expertise of physicians; safety; effectiveness; economics, organisational, social, and legal aspects	Need to improve the accuracy of cost estimates, incorporate patient preferences, include indirect costs, account for uncertainty, incorporate long‐term outcomes, and address equity concerns
	Cost‐effectiveness; health technology assessment [34, 37, 38]						
	Uncertainty; health technology assessment [34, 35, 36]						
Evidence‐based HTA in oncology	Oncology; real‐world evidence [8, 39, 40]	Using real‐world evidence data to assess oncology‐related drugs	Real‐world evidence methods working group (experts in HTA, health services research, statistical analysis, and health economics), decision‐makers/payers, HTA bodies, policymakers, patients, researchers, industry	1. Drug selection 2. Collection of data 3. Assessment Funding decision	Review, C/E analysis	Safety, clinical‐effectiveness, cost‐effectiveness	The need for more research on patient‐centred outcomes and on the use of real‐world data. How to appropriately and accurately conduct economic evaluations in this field, particularly in the context of emerging treatments such as immunotherapy.
HTA review‐based guidelines	Guidelines; economic evaluation [2, 13, 41]	Guidelines developing using reviews of economic evaluations	Expert panel (perspectives of analysis: Societal, government, patient, hospital, third party payer)		Targeted literature review (TLR) about C/E, C/U, C/B analysis; critical review of studies; cost‐minimisation analysis; budgetary impact analysis; review of utilization data; Outcomes: QALY, DALY, ICER, contingent valuation, Life‐years, clinical end points, monetary	Costs (unit prices, quantity of physical resources, direct health system costs, out‐of‐pocket expenditures, indirect costs) Utility (quality of well being scale, standard gamble and rating scale) Effectiveness (disease specific outcome that reflect success of therapy, disease‐specific rating scales, avoided hospitalisation) rather than efficacy Equity	The absence of a structured method to realise HTA evidence‐based guidelines that goes beyond the traditional literature review
	Guidelines; review [13, 42]						
Micro^1^‐meso^2^ HTA	Hospital‐based‐HTA; Quebec [43, 44]	1. Hospital‐based HTA related to Quebec health technology assessment. 2. CADTH activities for assessing orphan drugs for rare disease	1. Decision makers, HTA producers, HTA professionals, clinicians, patients, caregivers, citizens, researchers. 2. CADTH canadian drug expert committee (CDEC), HTA agencies, patients, clinicians, payers		1. Horizon scanning, reviews based on publications in scientific journal, health evaluation methods, mathematical decision model. 2. Cost‐utility analysis, cost‐consequence and cost‐minimisation analysis (markov model, decision tree, micro‐simulation model) Outcomes: QALY (50k–400k threshold), ICURs (incremental cost utility ratios)	1. Clinical effectiveness, cost effectiveness, social, ethical, and organisational impacts of technologies and interventions, safety, efficacy. 2. Cost effectiveness.	1. How to optimise resource allocation, overcome barriers to HTA implementation and adoption, and determine the impact and outcomes of hospital‐based HTA for demonstrating its value 2. How to join qualitative and quantitative considerations in the HTA procedures
	Hospital‐based‐HTA; health technology assessment [14, 45]						
	CADTH; orphan drugs; rare diseases [3]						
Macro‐HTA	Health policy; technology assessment [3, 46]	Macro‐HTA (health policy) activities related to technology assessment in biomedical field	Policymakers, HTA bodies, evidence synthesis experts, clinicians, pharmacists, providers, patients, industry, drug innovators	1. Scope, purpose, and deadline refinement phase 2. Literature searching 3. Study selection 4. Data abstraction 5. Evidence synthesis 6. Critical appraisal 7. Report production and dissemination	Rapid review (PICOS framework, PRISMA‐P formal research protocol) Traditional literature review Economics analysis (cost‐minimisation, cost‐utility, cost‐effectiveness, cost‐benefit analysis) Cost/financial impact (budget impact analysis) Canadian guidelines for evaluation	Technological features, safety, effectiveness, efficacy, ethical, social, and legal considerations, clinical outcomes, resource use and costs, product quality, IT specifications/quality, clinical trials, ease of use, environmental considerations, price, total cost, value‐adds	How to incorporate broader societal perspectives into macro‐level decision‐making, ensure greater consistency and transparency, and elicit patient preferences. To determine the actual impact of the macro‐HTA outputs on the lower HTA decision‐making.
	Health policy; biomedical [3, 47, 48, 49]						
	Reimbursement; decision‐making [48]						
Patient involvement	Patient involvement; health technology assessment [24, 50, 51]	Patient involvement/engagement in health technology assessment processes	General/interested public, patients, broader public/patients, authors of public comment submissions, policymakers, managers, researchers, citizens	1. Topic selection 2. Scoping 3. Evidence‐based analysis 4. Draft recommendation 5. Professional and public consultation 6. Assessment of comments 7. Post review and recommendation	Clinical and cost effectiveness analysis	Safety, clinical‐effectiveness, clinical benefit, cost‐effectiveness	The need for research to determine the most effective ways to engage patients throughout the HTA evaluations, for further research on how to incorporate patient preferences into decision‐making, and on how to measure the impact of patient involvement.
	Patient engagement; health technology assessment [52, 53, 54]						

### 4.4 Research Streams

#### 4.4.1 Ethics in HTA Processes

This RS focuses on integrating ethical criteria in technology evaluations [4, 6, 18] creating social value [6, 30, 32].

Ethics in HTA remains unevenly applied, limited by economic priorities and assumptions of value‐neutrality. Initiatives (e.g., EUNetHTA ethic domain) promote context‐aware ethical analysis but agreed standards and methods are still missing.

Key stakeholders include patients, Health Systems, clinicians, members of the Health Technology Expert Review Panel (HTERP) from Canada's Drugs and Health Technology Agency (CADTH), customers, experts, ethicists, HTA bodies, and policymakers [4, 18, 30]. Patients are critical as they are directly affected by technology outcomes. Health Systems and clinicians are essential for implementation and use, while HTA bodies and policymakers decide on technology approvals, considering ethical issues such as equity, justice, autonomy, and non‐maleficence. Their role ensures transparency and fairness in decision‐making. Diverse stakeholder involvement is crucial for ethical and socially responsible evaluation of technologies, maximising benefits to patients and society.

The evaluation phases align with those in the ax path.

Regarding methodologies [18], discusses CADTH's Deliberative Framework, a multi‐criteria tool evaluating 10 domains, including ethics and environmental impact. Multi‐criteria tools in this RS support a comprehensive assessment by incorporating ethical considerations alongside traditional criteria like cost‐effectiveness.

A research gap exists in the need for clear ethical frameworks to guide HTA decision‐making consistently. While this RS supports the use of MCDA for integrating both quantitative and qualitative criteria, especially ethical ones, the challenge persists in effectively merging these aspects within HTA processes, despite its recognized importance in the literature.

#### 4.4.2 Economic Analyses

This RS advocates using C/E and C/U analysis as primary methodologies under uncertainty [33, 34, 35, 36]. These analyses use ICER and QALY to assess healthcare costs and expected life years [37].

Process phases are two: evaluating impact in terms of life years and healthcare costs [20]. The criteria include measuring technology costs, utility, effectiveness [33, 37], and its direct effects on patients, clinical outcomes, management, physician expertise, and safety [34].

Technical stakeholders such as specialist groups, agencies, methodologists, healthcare managers, and programme directors are involved in these processes [36, 37, 38]. C/E analysis, requiring skills in economics and statistics, examines costs relative to benefits and outcomes to determine cost‐effectiveness. This demands expertise in the healthcare system, technology, and statistical methods.

While C/E analysis is widely used, it needs improvement in accuracy, patient preferences, indirect costs, managing uncertainty, long‐term outcomes, and equity concerns. Currently, it focuses mainly on economic outputs and often overlooks ethical and social aspects. Future research should integrate equity‐based considerations into C/E analysis.

#### 4.4.3 Evidence‐Based HTA in Oncology

This RS focuses on evidence‐based HTA for assessing oncology drugs [8, 39, 40]. Oncology is a key focus in HTA due to the high cost of cancer drugs and the research rapid advancements. These factors make it a critical area of study, with significant implications for healthcare system sustainability.

The RS overlaps with both the ethics and economic analyses ones, as it can be seen as a subset of each. Economic evaluations are considered due to high drug costs, while ethics remains central to decisions around procurement and reimbursement—especially when cost‐effectiveness alone would argue against adoption.

Stakeholders include HTA experts, health‐services researcher, statistics, and health economics [40], along with decision‐makers, payers, HTA bodies, policymakers, patients, and industry [8]. The phases of the process are [8]: drug selection, data collection, assessment, and funding decision. Methodologies used are literature reviews and C/E analysis, with evaluation criteria including safety, clinical‐effectiveness, and cost‐effectiveness [8, 40].

Gaps include a need for more studies on patient‐centred outcomes such as quality‐of‐life, symptom relief, and patient satisfaction, the use of real‐world data, and a deeper analysis of drug cost criteria. Research should focus on improving economic evaluations in this field, particularly for emerging treatments like immunotherapy.

#### 4.4.4 HTA Review‐Based Guidelines

This RS focuses on developing HTA guidelines through literature reviews. The primary articles reviewed for this process are economic evaluations of biomedical technologies [2, 13, 41, 42].

Key stakeholders are HTA expert panels (healthcare providers, statisticians, and economists) responsible for synthesising evidence and making recommendations [13, 42]. Panels may also involve patients, healthcare providers, and industry representatives for input and feedback.

Methodologies used in this process include Targeted Literature Reviews (TLR) [31], specifically designed to review previous economic analyses, such as C/E, C/U, and cost‐benefit (C/B), focusing on outcomes like ICER and QALY, and criteria such as technology costs, utility, effectiveness, and equity [2, 13, 41]. Literature reviews are crucial for developing HTA guidelines as they ensure evidence‐based recommendations.

Research gaps include the need for standardized HTA methodologies, better evaluation of study quality, applicability across diverse settings, and integration of non‐traditional data sources like patient‐reported outcomes. A need for a structured approach to developing HTA guidelines for medical devices, which currently lacks due to limited evidence compared to drug‐related literature, emerges.

#### 4.4.5 Micro–Meso HTA

This RSs encompasses two topics: Micro‐HTA and Meso‐HTA.

Micro‐HTA (hospital‐based HTA) focuses on local evaluations of individual technologies/treatments, particularly in Quebec [14, 43, 44, 45]. Stakeholders include local decision‐makers, HTA producers and professionals, clinicians, patients, caregivers, citizens, and researchers. Methodologies include Horizon‐Scanning, reviews, and mathematical decision models [43, 44]. Evaluation criteria commonly include clinical/cost‐effectiveness, as social, ethical, and organizational impacts, safety and efficacy. Research gaps include optimising resource allocation to maximise patient benefit, overcoming barriers to HTA implementation, involving clinicians in HTA processes, and demonstrating the hospital‐based‐HTA value in decision‐making and patient‐outcomes.

Meso‐HTA pertains to provincial assessments conducted by CADTH, particularly for orphan drugs [3]. In this context, stakeholders include the expert committees, agencies, patients, clinicians, and payers. Monetary methodologies, such as C/U analysis, cost‐consequence, and cost‐minimisation analysis are employed. The limitations involve integrating qualitative criteria into mathematical decision models. Future research should address how to effectively combine qualitative and quantitative considerations in HTA.

#### 4.4.6 Macro‐HTA

This RS focuses on Macro‐HTA, aiming to assess biotechnologies for recommendation reports, supporting the reimbursement process, and evaluating health policies impact [3, 46, 47, 48].

The stakeholders involved are policymakers, regional bodies, evidence synthesis experts, clinicians, pharmacists, providers, patients, drug innovators, and industry [46, 48, 49].

Macro‐HTA reports are used to conduct evaluations at meso/micro‐HTA levels regarding the comparison between technologies. Macro‐HTA process is more structured and includes several phases [46]: scope, purpose, and deadline refinement; literature searching; study selection; data abstraction; evidence synthesis; critical appraisal; and report production and dissemination.

The methodologies used include rapid reviews performed using different frameworks and formal protocols, traditional literature reviews, economics, costs, and financial impact analysis [3, 46, 48]. The evaluation criteria encompass technical (e.g., technological features, IT quality, safety), clinical (e.g., clinical outcomes and trial), practical (e.g., ease of use and product quality), monetary (e.g., resource use, costs, and price), and organizational and sustainability aspects (e.g., environmental, ethical, social considerations, and value‐adds) [3, 47, 48, 50].

Research gaps in Macro‐HTA involve integrating broader societal perspectives into decision‐making, enhancing consistency and transparency to minimise bias, and incorporating patient preferences into macro‐level choices. Future research should assess the tangible impact of Macro‐HTA on meso/micro‐level decision‐making. Despite being utilised to evaluate technology selection for clinical practice, there is a lack of understanding regarding the actual influence of these outputs on lower‐level HTA decisions.

#### 4.4.7 Patient Involvement

This RS addresses the growing importance of patient involvement in the HTA processes [51, 52, 53, 54]. Unlike the b1 path, this RS emphasises patient involvement at the macro‐HTA level.

Articles detail several phases of the macro‐HTA processes [50, 51]: topic selection, scoping phase, evidence‐based analysis, draft recommendations, professional and public consultations, assessment of comments, and post‐review recommendations production.

Stakeholders include patients, citizens, policymakers, health managers, and researchers [50, 51]. Methodologies used are clinical and C/E analysis, with evaluation criteria covering safety, clinical benefit, and cost‐effectiveness.

Research gaps include identifying optimal methods for patient engagement throughout the evaluation process, integrating patient preferences in decision‐making amidst uncertainty or conflicting evidence, and assessing the impact of patient involvement on outcomes (e.g., decision‐making quality, patient‐centred care) and processes (e.g., trust, engagement, satisfaction among patients and stakeholders).

As summary, Figure 6 illustrates the RSs identified.

**FIGURE 6:**
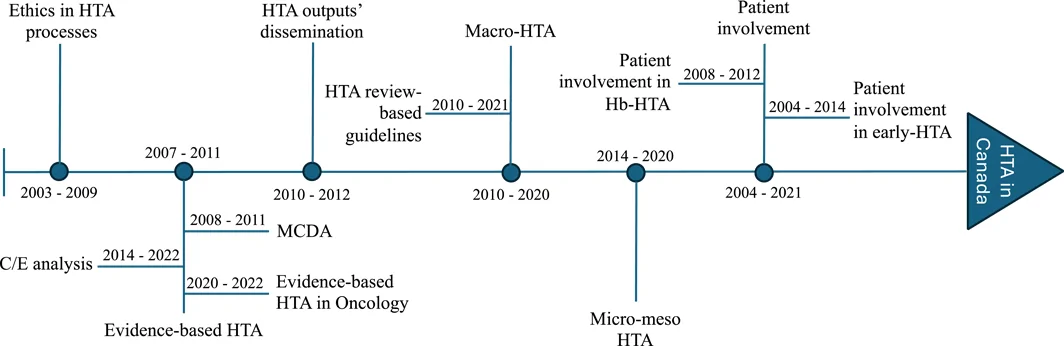
Summary.

## 5 Discussion and Contribution

This paper had three main objectives: firstly, to highlight the most investigated Canadian HTA RSs through a SLNA; secondly, to identify the key elements of the HTA process in these RSs, including stakeholders, phases, methodologies, and evaluation criteria; and thirdly, to identify literature gaps and suggest new research directions.

Consistently with previous reviews, HTA in Canada is a complex, decentralised process, and encompasses various healthcare fields and levels. The research identifies several prominent RSs within the Canadian HTA literature (RQ1). Table 3's first three columns put forward the name, the keywords, and the descriptions of each RSs: ethical considerations in HTA, economic analyses, evidence‐based HTA in oncology, HTA review‐based guidelines, Micro‐Meso‐HTA, Macro‐HTA, and patient involvement. This comprehensive overview surpasses previous studies focused on specific HTA aspects in Canada, aligning with the primary goal of literature reviews to provide an exhaustive perspective.

Analysing the RSs through the organizational processes' lens revealed that each RS is shaped by distinct elements (RQ2) that define its internal structure, contributing to strong internal coherence, while limiting connections with other RSs. Columns 4 through 7 of Table 2 outline these elements.

A primary distinction among RSs is the stakeholder variety, which directly influences the assessment approach, leading to varying evaluation perspectives. For example, ethical‐HTA primarily involves ethicists, policymakers, and HTA bodies, while economic analyses are driven by health economists, HTA agencies, and healthcare managers (e.g., [33, 34, 37]). Oncology‐HTA engages experts in real‐world evidence, pharmaceutical industry representatives, and decision‐makers, whereas macro‐HTA is focused on broad policy decisions, involving policymakers and industry stakeholders. Additionally, patient involvement HTA integrates patient, caregiver, and citizen opinions (e.g., [14, 30, 43]).

The process phases also vary significantly among RSs. Economic analyses typically follow a two‐step structure, assessing life expectancy impact and healthcare costs, while ethical HTA follows a three‐phase decision‐making process, including reviewing past decisions, addressing new cases, and integrating decisions into future frameworks. Oncology‐HTA follows a more pragmatic sequence, beginning with drug selection, data collection, and funding decisions. Macro‐HTA is structured around systematic evidence synthesis, including literature searches, critical appraisals, and policy‐focused reporting (e.g., [3, 46]). In contrast, micro‐HTA lacks formalised phases, for example, in hospital‐based HTA, where the process is less structured (e.g., [14, 45]).

Methodologies further differentiate the RSs. Economic analyses rely heavily on C/E, using metrics like QALY and ICER, while ethical‐HTA employs quali‐quantitative frameworks. Oncology‐HTA uniquely integrates real‐world evidence, making it distinct from traditional clinical trial‐based assessments. Macro‐HTA applies rapid reviews and economic modelling to inform large‐scale healthcare policies. In some RSs, hybrid methods are used, combining quantitative and qualitative criteria, including mathematical, review‐based, and economic methods (e.g., [13, 14, 33, 34]). Patient involvement HTA also employs these methodologies to reflect patient preferences in QALY metrics (e.g., [24, 52]).

Evaluation criteria show further differences among RSs. Economic analyses emphasise direct patient benefits, clinical outcomes, and cost‐utility considerations (e.g., [33, 39]), while Oncology‐HTA incorporates real‐world safety and effectiveness data. Macro‐HTA broadens the evaluation scope to include social, ethical, and regulatory aspects, while patient involvement HTA focuses on assessing safety and clinical‐effectiveness (e.g., [14, 42]).

Despite the independence of each RS and their strong internal consistency, some overlap exists. For example, economic analyses contribute to the development of HTA‐based guidelines, while macro‐HTA influences decision‐making at micro‐ and meso‐levels. Despite these intersections, each RS remains largely self‐contained, as the processes, methodologies, and objectives vary significantly. This creates a complex yet structured HTA landscape, where different RSs address distinct aspects of health‐technology evaluation while maintaining limited cross‐integration.

Moreover, this study extends beyond the literature reviews that focused on HTA impact on a healthcare system [21]. Through this comprehensive overview of HTA process elements, we aim to highlight the need to break down the HTA process into its components, since each of them contributes to the overall process performance, thus to the value creation towards users.

Column 8 of Table 3 identifies key literature gaps (RQ3), including the integration of qualitative and quantitative considerations (e.g., [4, 6, 30]), the need for equity‐based cost‐effectiveness analyses (e.g., [34, 37]), the development of structured HTA guidelines methodologies (e.g., [30, 31]), and the assessment of the impact of the macro‐HTA outputs on meso‐ and micro‐HTA evaluations. Overcoming barriers to patient involvement is also critical (e.g., [5, 19, 24]).

To address these gaps and guide future research, new qualitative studies are recommended. Indeed, the most effective methodologies for investigating phenomena in fields with limited knowledge (such as the one examined in this work) are those that collect data from individuals directly experiencing the phenomenon ‘within its real‐life context’. Examples of these methodologies include focus groups with HTA experts, expert and elite interviews, and case studies.

Theoretically, by mapping Canadian HTA through citation links, this study is the first to adopt a process perspective for analysing differences in RSs regarding HTA. Additionally, it highlights future research challenges, contributing to a deeper understanding of how HTA is structured and evolves in Canada. By applying a systematic approach to categorise RSs and their interconnections, this research provides a foundational framework for future studies exploring HTA processes.

Practically, this study provides HTA practitioners with useful information regarding the key elements of the HTA processes. By delineating stakeholder roles, methodological variations, and evaluation criteria, the study facilitates a clearer understanding of the decision‐making mechanisms within HTA. These insights can support professionals in designing more effective and inclusive HTA processes. Moreover, identifying literature gaps and proposing methodological advancements can help refine HTA guidelines, enhancing their applicability in diverse healthcare settings.

From the policy perspective, several recommendations emerge from the study. Policymakers should ensure comprehensive stakeholder engagement in HTA evaluations, both in terms of breadth (diversity of stakeholder types) and depth (intensity of involvement). Effective HTA requires not only involving a range of stakeholders but also integrating their feedback appropriately throughout the HTA process. This approach will enhance the rigour and fairness of decision‐making and support more transparent and inclusive HTA policies. Additionally, addressing literature gaps suggests several policy actions: standardising HTA processes across countries to ensure consistency, prioritising assessments of technologies with significant clinical and economic impacts, and investing in research to refine HTA methodologies, data collection, and long‐term outcome evaluations.

### 5.1 Limitation

The study's focus on Canadian HTA may limit the generalisability of the results. It provides an overview of Canadian HTA elements across different RSs but does not assess the effectiveness of various HTA methodologies or criteria in detail. As a literature review, it is constrained by the availability and quality of existing research.

## Funding

The research was developed with the support of funds from THE—Tuscany Health Ecosystem (Spoke 5)—CUP—I53C22000780001—PNRR MISSIONE 4—COMPONENTE 2—ECOSISTEMI PER L’INNOVAZIONE.

## Ethics Statement

The authors have nothing to report.

## Conflicts of Interest

The authors declare no conflicts of interest.

## Data Availability

Data sharing not applicable to this article as no datasets were generated or analysed during the current study.
